# A second-hit conceptual framework for pulmonary arterial hypertension in adult congenital heart disease: genetics, hemodynamics, and treat-and-repair

**DOI:** 10.3389/fcvm.2026.1907009

**Published:** 2026-08-04

**Authors:** Daniel Alejandro Del Salto-Campuzano, Carol Marcelle García-Chehab, Martín Campuzano-Donoso, Claudia Reytor-González, Daniel Simancas-Racines

**Affiliations:** 1 Independent Researcher, Quito, Ecuador; 2 Center for Evidence Ecosystems, Implementation Science, and Decision-Making (CIDES), Facultad de Ciencias de la Salud y Bienestar Humano, Universidad Tecnológica Indoamérica, Ambato, Ecuador

**Keywords:** 4D-Flow MRI, adult congenital heart disease, endothelial-to-mesenchymal transition, Fontan circulation, pulmonary arterial hypertension, second-hit framework, SOX17, treat-and-repair

## Abstract

Pulmonary arterial hypertension (PAH) complicates a clinically important subset of adult congenital heart disease (ACHD) and contributes to substantial morbidity and premature mortality. This narrative review synthesizes direct and indirect evidence within a second-hit conceptual framework for ACHD-associated PAH. In this framework, baseline pulmonary vascular susceptibility interacts over time with sustained hemodynamic or hypoxemic exposure and with superimposed biological or clinical stressors. SOX17 has the clearest direct genetic association with ACHD-PAH, whereas evidence involving BMPR2-related metabolic, inflammatory, and proliferative pathways is derived predominantly from heritable PAH, broader PAH populations, and experimental models. Experimental and computational studies also suggest that disturbed flow and mechanobiological signaling may contribute to endothelial dysfunction and endothelial-to-mesenchymal transition, although these mechanisms have not been established as a causal sequence in longitudinal ACHD cohorts. Four-dimensional flow magnetic resonance imaging and cardiopulmonary exercise testing may characterize abnormal flow patterns and impaired pulmonary vascular reserve in research settings, but neither is validated for screening patients with borderline ACHD hemodynamics or for selecting therapy. Evidence supporting treat-and-repair remains observational and defect-specific, with the most developed adult data derived from carefully selected patients with atrial septal defects; these findings should not be extrapolated directly to ventricular septal defects, patent ductus arteriosus, or complex congenital heart disease. Fontan physiology is considered separately as a low-flow, low-reserve pulmonary vascular state rather than as classical high-pressure PAH. The principal contribution of this review is to organize heterogeneous evidence according to its directness, distinguish established clinical practice from investigational approaches, and identify priorities for prospective validation. The second-hit framework should therefore be interpreted as a hypothesis-generating conceptual synthesis rather than as a validated diagnostic or therapeutic algorithm.

## 1 Introduction

The growing population of adults living with congenital heart disease (ACHD) has increased the complexity of long-term cardiovascular care, with surveillance of late complications becoming a central priority as survival improves (1–4). Pulmonary arterial hypertension (PAH) remains a major contributor to morbidity and mortality, including in patients with repaired congenital lesions (1, 5, 6). Approximately 4%–10% of adults with congenital heart disease are estimated to develop ACHD-associated PAH (ACHD-PAH), although prevalence varies by cohort, lesion type, and repair status (1, 7–9). This distribution is evolving, as earlier surgical repair has reduced the frequency of classic Eisenmenger physiology, while postoperative pulmonary hypertension is increasingly recognized in patients with complex or repaired lesions (1, 10). Together, these changes may suggest that anatomy alone cannot explain why some ACHD patients develop pulmonary vascular disease while others with similar defects do not.

ACHD-PAH has traditionally been interpreted largely through an anatomical and hemodynamic lens, particularly in patients with unrepaired or incompletely repaired left-to-right shunts that chronically expose the pulmonary vasculature to increased flow and pressure. Historically, Eisenmenger syndrome represented the advanced, often irreversible end of PAH associated with congenital heart disease, whereas contemporary strategies place greater emphasis on earlier recognition, risk stratification, and prevention of progression (6, 11). The 2022 European Society of Cardiology/European Respiratory Society (ESC/ERS) guidelines further lowered the hemodynamic threshold for diagnosing pulmonary hypertension (12). Pre-capillary pulmonary hypertension is now defined as mean pulmonary arterial pressure (mPAP) >20 mmHg, pulmonary arterial wedge pressure (PAWP) ≤15 mmHg, and pulmonary vascular resistance (PVR) >2 Wood units, replacing the historical thresholds of mPAP ≥25 mmHg and PVR ≥3 Wood units (12). These revised thresholds bring attention to patients with mildly abnormal or borderline hemodynamics, such as mPAP 21–24 mmHg with PVR between 2 and 3 Wood units, in whom closer surveillance may be appropriate, but the efficacy of PAH-targeted therapy in this subgroup remains unknown (12). Current PAH management emphasizes earlier identification and multipathway therapy, although prognosis, treatment response, and the strength of evidence vary across PAH subgroups (13).

Beyond differences across PAH subgroups, marked heterogeneity is observed among ACHD patients with apparently comparable anatomical defects. Anatomy alone therefore does not fully explain why patients with similar lesions may follow markedly different pulmonary vascular trajectories. For the purposes of this review, the “first hit” denotes baseline pulmonary vascular susceptibility related principally to genetic, developmental, or endothelial factors. The “second hit” denotes additional exposures that may amplify this susceptibility. We distinguish sustained second hits, such as chronic high pulmonary flow, pressure loading, abnormal shear stress, or hypoxemia, from superimposed second hits, such as infection, atrial arrhythmia, pregnancy, or acute inflammatory or metabolic stress. These categories are conceptual rather than prospectively validated, and the relative contribution of each exposure is likely to differ according to defect anatomy, repair status, age at repair, and cumulative duration of exposure (6, 12, 14–17). The second-hit concept is therefore used here as an organizing hypothesis rather than as a new disease classification or clinical pathway.

We aim to provide an integrated narrative synthesis of ACHD-PAH as a biologically heterogeneous process by examining evidence across genetic, molecular, hemodynamic, imaging, therapeutic, and multiorgan domains. Rather than proposing a diagnostic or therapeutic algorithm, we evaluate how these domains may help explain divergent pulmonary vascular trajectories, distinguish evidence that is directly derived from ACHD-PAH from evidence extrapolated from other populations or experimental models, and identify questions requiring prospective validation. Throughout the review, established clinical recommendations are separated from mechanistic hypotheses and investigational applications.

## 2 Methods

This article was designed as a narrative review with a structured, purposive literature search and evidence-informed synthesis. A narrative design was selected because the objective was to integrate heterogeneous genetic, molecular, hemodynamic, imaging-based, pharmacological, and multiorgan evidence into a mechanistic framework for understanding pulmonary arterial hypertension associated with adult congenital heart disease (ACHD-PAH), rather than to answer a narrowly framed intervention, diagnostic-accuracy, or prognostic question. The review therefore did not aim to estimate pooled treatment effects, perform certainty grading, or generate a systematic-review evidence map.

Accordingly, this review was not registered as a systematic review, no PRISMA flow diagram was constructed, and no formal systematic-review procedures were undertaken, including duplicate independent screening, exhaustive database searching, formal risk-of-bias assessment, certainty-of-evidence grading, or quantitative meta-analysis. The manuscript was prepared with reference to reporting principles reflected in the Scale for the Assessment of Narrative Review Articles (SANRA), particularly justification of the review's importance, clear statement of aims, description of the literature-search approach, appropriate referencing, and scientific reasoning (18). However, no formal item-level SANRA scoring was performed.

A structured PubMed search was conducted in April 2026 to identify peer-reviewed literature relevant to the second-hit framework. PubMed was selected as the primary database because it provides broad biomedical indexing of pulmonary vascular disease, congenital heart disease, cardiovascular imaging, exercise physiology, and clinical therapeutics. The search combined Medical Subject Headings (MeSH) and free-text terms across four prespecified thematic domains: genetic susceptibility and endothelial biology; multimodal hemodynamic and exercise-based assessment; PAH-targeted therapy, operability, and defect closure; and Fontan physiology with systemic complications. Terms within each domain were combined using OR, and domains were combined using AND when cross-domain specificity was required. Search terms and representative query structures are provided in Supplementary Tables S1, S5.

English-language peer-reviewed publications from 2019 to 2026 were prioritized. Foundational publications from 2018 to 2020 and selected earlier guideline or seminal references were retained when essential for clinical definitions, operability thresholds, genetic or mechanistic interpretation, or congenital heart disease context. Eligible source types included clinical guidelines, consensus statements, randomized trials when available, prospective and retrospective observational studies, registry analyses, systematic reviews, high-quality narrative reviews, and mechanistic, experimental, or computational studies relevant to the conceptual model. Preprints, non-peer-reviewed materials, and publications without clear relevance to ACHD-PAH, pulmonary vascular pathobiology, Fontan physiology, hemodynamic imaging, exercise hemodynamics, or PAH-targeted therapy were excluded or deprioritized.

Articles were selected purposively according to thematic relevance, methodological credibility, clinical applicability, and contribution to the mechanistic or conceptual scope of the review. Record counts were not prospectively logged; therefore, the numbers of retrieved, screened, excluded, and included articles are not reported. This is consistent with the narrative design but is acknowledged as a limitation. Evidence was interpreted according to its directness in relation to ACHD-PAH. Direct evidence was defined as evidence derived from adult congenital heart disease populations with PAH, Eisenmenger syndrome, shunt-associated PAH, or repaired congenital lesions. Evidence from Fontan cohorts was considered directly relevant to Fontan physiology but indirect when used to inform classical ACHD-PAH. Partially direct evidence included mixed-age or mixed-congenital-heart-disease cohorts in which ACHD-PAH-relevant findings could be interpreted but were not isolated. Indirect evidence included broader PAH cohorts, pediatric congenital heart disease or pediatric PAH cohorts, experimental models, computational hemodynamic studies, and non-ACHD populations used to support biological plausibility.

Greater interpretive weight was given to clinical guidelines, adult ACHD cohorts, prospective studies, large observational series, and systematic reviews when available. Mechanistic, imaging-based, computational, pediatric, and non-ACHD data were used primarily to support biological plausibility, identify knowledge gaps, or generate hypotheses for future ACHD-specific research. When evidence was extrapolated from non-ACHD populations or from mechanistic studies, conclusions were framed as hypothesis-generating rather than as established clinical recommendations. The resulting synthesis was interpreted according to the directness of the available evidence and was not intended to constitute a clinical practice guideline.

## 3 Genetic and molecular basis of the second-hit framework: SOX17, BMPR2, endothelial plasticity, and shear stress

### 3.1 The genetic predisposition

Genetic predisposition may act as a first hit in pulmonary vascular susceptibility. More than ten genes show definitive evidence as causal for pulmonary arterial hypertension overall (15), although the strength of direct evidence in ACHD-PAH varies across genes. Among the genes discussed in this context, SOX17 has the clearest direct association with ACHD-PAH cohorts, whereas evidence involving BMPR2 is derived predominantly from heritable PAH, broader PAH populations, and experimental models. Additionally, susceptibility genes including TBX4, FOXF1, and KCNK3 are established for PAH broadly but with more limited direct evidence in adult congenital populations (Table 1). This susceptibility architecture overlaps substantially with that of heritable PAH, particularly through BMPR2-related pathways (19, 20). BMPR2, encoding the type II receptor of the bone morphogenetic protein (BMP) signaling axis, is the most frequently mutated gene in heritable PAH, accounting for approximately 75% of cases in affected families (19, 20). In sporadic and CHD-associated PAH, BMPR2 variants are identified in a smaller minority, and direct evidence for their pathogenic role specifically in ACHD-PAH remains more limited than in heritable forms (19, 21). Experimental evidence suggests that BMPR2 haploinsufficiency may impair the ability of pulmonary arterial cells to maintain vascular quiescence, thereby increasing vulnerability to abnormal hemodynamic stress and chronic shunt-related pulmonary blood flow (19, 21, 22). These findings support biological plausibility for a susceptibility pathway, but do not establish BMPR2 as a common or primary cause of ACHD-PAH (19). Accordingly, BMPR2-related mechanisms are considered here as models of pulmonary vascular susceptibility rather than as evidence that BMPR2 dysfunction is a dominant driver of ACHD-PAH.

**Table 1:** Selected genes relevant to PAH susceptibility and the second-hit framework in congenital heart disease-associated PAH.

Gene	Main biological role	Relevance to ACHD-PAH	Directness of evidence in ACHD-PAH	Key references
BMPR2	BMP signaling; vascular quiescence	Most frequently mutated gene in heritable PAH; variants have been reported in a small minority of CHD-PAH cases, while experimental BMPR2 dysfunction provides a model of pulmonary vascular susceptibility to additional hemodynamic or biological stress.	Predominantly indirect—derived from heritable PAH, broader PAH cohorts, and experimental BMPR2 models; direct ACHD-PAH genetic evidence is limited, and BMPR2 is not established as a common driver in this population.	(19–22)
SOX17	Endothelial identity and pulmonary vascular development	Rare deleterious variants identified in approximately 3% of ACHD-PAH cases; frequent association with congenital cardiac anomalies	Direct - derived from ACHD-PAH cohort sequencing studies	(14, 15, 23–25)
TBX4/T-box genes	Cardiopulmonary morphogenesis	Rare T-box variants reported in adults with ACHD-PAH; substantial evidence base derives from pediatric PAH	Direct but limited - adult ACHD-PAH data are smaller in scale; pediatric and experimental data predominate	(15, 27, 28)
FOXF1	Endothelial integrity and DNA-damage response	Reduced FOXF1 may amplify endothelial vulnerability, particularly in BMPR2-deficient settings	Indirect - predominantly experimental and broader PAH evidence; ACHD-PAH-specific data limited	(15, 22)
KCNK3	Potassium channel; pulmonary vascular tone	Susceptibility gene established for heritable and idiopathic PAH; mechanistic plausibility in ACHD-PAH	Indirect - heritable/idiopathic PAH cohorts; minimal ACHD-PAH-specific evidence	(15, 20, 24)

The listed genes differ in the strength and directness of evidence for ACHD-PAH. Some associations derive from heritable or idiopathic PAH cohorts, broader PAH gene-validity studies, or experimental models and should be interpreted as supporting susceptibility or mechanistic plausibility rather than ACHD-specific causality. ACHD, adult congenital heart disease; ACHD-PAH, pulmonary arterial hypertension associated with adult congenital heart disease; BMP, bone morphogenetic protein; BMPR2, bone morphogenetic protein receptor type 2; CHD, congenital heart disease; PAH, pulmonary arterial hypertension; SOX17, SRY-related HMG-box transcription factor 17; TBX4, T-box transcription factor 4.

Genome-wide and panel-based studies have identified SOX17 as the PAH susceptibility gene with the clearest direct association in ACHD-PAH cohorts, with rare deleterious variants identified in approximately 3% of ACHD-PAH cases (15, 23, 24). SOX17 (SRY-related HMG-box transcription factor 17) regulates endothelial identity and pulmonary vascular development, and emerging data suggest interactions with sex-related and metabolic pathways (25). Lacoste-Palasset et al. synthesized the developmental biology of SOX17 in PAH, from embryonic vascular specification to adult clinical phenotypes, and highlighted that SOX17-associated disease frequently develops in the setting of congenital cardiac anomalies (14, 23). In experimental models, Mahomed et al. showed that SOX17 silencing upregulates NF-κB-induced CXCL10 and CXCL11, linking SOX17 loss to an inflammatory endothelial phenotype relevant to vascular remodeling (26). These observations support the possibility that SOX17 insufficiency may increase endothelial vulnerability before clinically overt hemodynamic deterioration becomes apparent (14, 26).

The clinical implication of these genetic factors is that ACHD patients with similar anatomical defects may not carry equivalent PAH risk. Gene panel diagnostics covering these susceptibility genes identify pathogenic or likely pathogenic variants in a subset of heritable PAH and in some CHD-PAH cases (20, 27) (Table 1). Li et al. reported rare T-box variants in adults with ACHD-PAH, supporting non-anatomical genetic contributors to disease susceptibility (28). Genetic counseling for relatives of patients with pathogenic PAH variants is recommended by the 2022 ESC/ERS guidelines (12). However, the value of genetic testing for predicting PAH development or progression in unselected ACHD patients, including those with borderline hemodynamics, remains unresolved.

Most mechanistic evidence linking BMPR2 dysfunction to metabolic, inflammatory, and proliferative remodeling derives from heritable PAH and experimental models rather than from ACHD-PAH cohorts. In these settings, BMPR2 loss-of-function has been associated with hyperglycolytic reprogramming and HIF-mediated transcriptional changes in pulmonary artery smooth muscle and endothelial cells, while TNF-α-mediated BMPR2 suppression, FOXF1 downregulation secondary to unrepaired DNA damage, and altered smooth-muscle BMPR2 signaling have also been implicated (19, 22, 29). These mechanisms provide biological plausibility for interactions between impaired BMP signaling and hemodynamic, hypoxic, or inflammatory stress. However, they have not been directly demonstrated as mediators of disease initiation or progression in ACHD-PAH cohorts and should therefore be interpreted as extrapolated mechanistic evidence rather than as an established ACHD-specific pathway.

### 3.2 Endothelial-to-mesenchymal transition as a cellular mediator of remodeling

One cellular mechanism that may link genetic susceptibility and hemodynamic stress to pulmonary vascular remodeling is endothelial-to-mesenchymal transition. EndMT is characterized by partial loss of endothelial identity and acquisition of mesenchymal features in cells of endothelial origin, and has been proposed as a potential contributor to structural pulmonary vascular remodeling, largely on the basis of experimental and general PAH evidence (16, 30, 31). In the pulmonary vasculature, EndMT may reduce endothelial quiescence and barrier function and contribute to intimal and medial remodeling, though direct longitudinal demonstration in ACHD-PAH cohorts is currently lacking (16, 31).

TGF-β/SMAD signaling has been implicated as a major pathway that may promote EndMT in experimental PAH models, and pharmacological interruption of this axis may attenuate EndMT markers and PAH-like features in preclinical systems (16, 30, 32). Disturbed flow has also been linked to EndMT through KLF2-mediated regulation of endothelial quiescence in experimental pulmonary hypertension (33). These mechanistic observations provide a biologically plausible link between hemodynamic stress and endothelial phenotypic change, but their direct relevance to ACHD-PAH progression remains to be demonstrated.

The scope of endothelial dysfunction in PAH extends beyond EndMT. Reviews of PAH endothelial biology have catalogued multiple dysfunctional axes, including inflammatory activation, metabolic shift, and oxidative stress, each of which may contribute to vascular remodeling (16, 17). Accordingly, the available evidence supports EndMT as a biologically plausible component of the proposed framework rather than as an established causal intermediate in ACHD-PAH. Endothelial biomarkers and imaging signatures associated with these pathways remain investigational and are not currently validated for ACHD-PAH risk stratification (16, 17, 32).

### 3.3 Shear stress as a hemodynamic second hit

A hemodynamic second hit may operate through mechanobiological pathways that connect the physical properties of pulmonary blood flow to intracellular signaling. Hiepen, Jatzlau, and Knaus framed this concept explicitly for BMP/TGF-β-related vascular disorders, arguing that biomechanical stress may act as a second hit that promotes remodeling in a genetically primed vascular wall (34). In congenital heart defects with left-to-right shunting, the pulmonary arteries are exposed to chronically elevated flow and velocity, creating a hemodynamic environment that may favor endothelial dysfunction and remodeling (8, 10). Shinohara et al. provided an experimental link between pathological shear and EndMT: using computational modeling and *in vitro* systems, they reported that high shear stress reduced ERG expression and promoted endothelial-mesenchymal reprogramming under the experimental conditions studied (35). This experimental mechanism may be relevant to ACHD phenotypes exposed to sustained high-flow or high-pressure pulmonary circulation, particularly post-tricuspid shunts, but it has not been validated longitudinally in patients with ACHD-PAH. Large unrepaired ventricular or atrioventricular septal defects may generate pulmonary arterial flow and shear patterns that overlap with ranges examined in experimental or computational studies, although equivalence between these model-derived conditions and human ACHD-PAH has not been established (35, 36).

Endothelial mechanotransduction involves multiple, partly overlapping sensor systems rather than a single mechanoreceptor. In systemic endothelial models, a junctional complex comprising platelet endothelial cell adhesion molecule-1 (PECAM-1), vascular endothelial cadherin (VE-cadherin), and vascular endothelial growth factor receptors transmits flow-related mechanical forces to downstream kinase and integrin-dependent signaling (37, 38). The mechanosensitive ion channel PIEZO1 provides an additional route through which membrane deformation and shear stress can induce calcium influx and regulate flow-responsive transcriptional programs, including KLF2 and KLF4 (39). In pulmonary vascular studies, PIEZO1 expression was increased in pulmonary arterial endothelial cells from patients with idiopathic PAH and in experimental PH, and PIEZO1-related signaling was also implicated in an experimental rat model of high-flow and high-shear pulmonary hypertension (40, 41). These findings support the plausibility that junctional complexes, ion channels, integrins, and downstream transcriptional programs participate in pulmonary endothelial flow sensing. However, their relative contribution to ACHD-PAH has not been established, and the available evidence should not be interpreted as demonstrating an ACHD-specific mechanotransduction pathway.

The transition from adaptive to maladaptive pulmonary vascular responses may involve vortical flow, recirculation zones, low-wall-shear regions, and oscillatory shear, although the clinical thresholds at which these patterns become injurious remain undefined. Szafron et al. developed a computational growth-and-remodeling framework for pulmonary arterial hemodynamics that captures the transition between adaptive and maladaptive responses of the vascular wall, illustrating within a computational framework how an initially compliant response can progress toward stiffness, medial hypertrophy, and intimal proliferation (36). Walter et al. extended this line of reasoning with a modeling study of hemodynamic unloading of the distal pulmonary circulation in PH, suggesting within a modeling framework that distal vessels may be exposed not only to elevated pressure but also by pulse and wave effects from the remodeled proximal vasculature (42). Smooth muscle cell contributions to flow- and hypoxia-associated remodeling have also been investigated. Klouda et al. implicated Cxcl12 in vascular remodeling under flow and hypoxic conditions in experimental pulmonary hypertension, extending the mechanobiological injury beyond the endothelial compartment to the medial layer (43).

**Figure 1:**
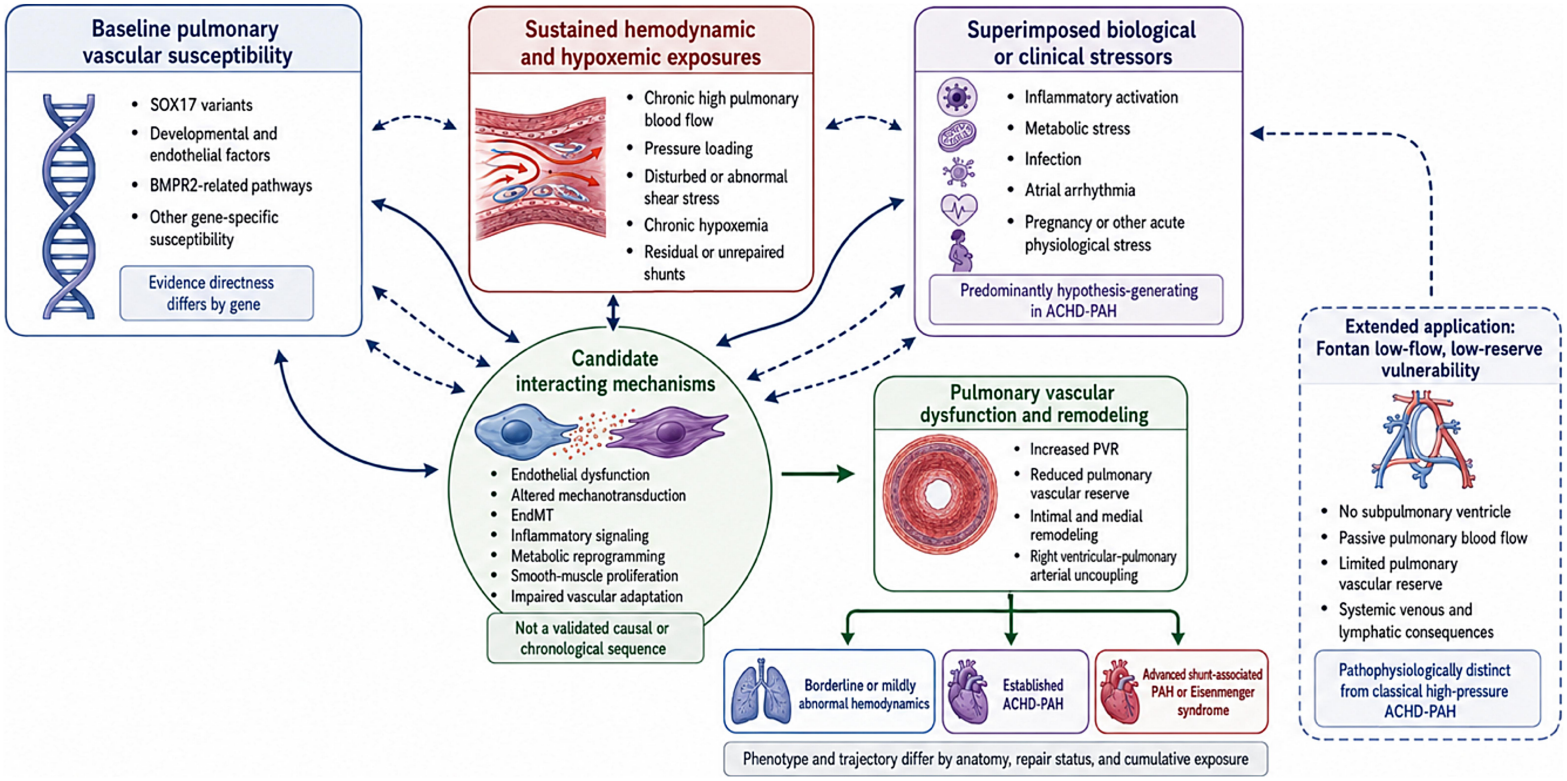
Conceptual and non-chronological second-hit framework in ACHD-PAH. Baseline pulmonary vascular susceptibility may interact with sustained hemodynamic or hypoxemic exposures and with superimposed biological or clinical stressors. These factors may converge through candidate mechanisms, including endothelial dysfunction, altered mechanotransduction, endothelial-to-mesenchymal transition, inflammatory signaling, metabolic reprogramming, smooth-muscle proliferation, and impaired vascular adaptation. The relationships shown are conceptual and potentially bidirectional and should not be interpreted as a prospectively demonstrated causal or chronological sequence in ACHD-PAH. Pulmonary vascular phenotypes may differ according to defect anatomy, repair status, age at repair, cumulative exposure, and other patient-specific factors. Fontan circulation is depicted separately as a low-flow, low-reserve state that is pathophysiologically distinct from classical high-pressure ACHD-PAH and Eisenmenger syndrome (1–17, 19–33, 44, 45). ACHD, adult congenital heart disease; ACHD-PAH, pulmonary arterial hypertension associated with adult congenital heart disease; BMPR2, bone morphogenetic protein receptor type 2; EndMT, endothelial-to-mesenchymal transition; PAH, pulmonary arterial hypertension; PVR, pulmonary vascular resistance; SOX17, SRY-box transcription factor 17.

Within the proposed framework, flow-mediated shear stress may represent both a marker of altered pulmonary hemodynamics and a potential contributor to endothelial dysfunction, although its independent role in ACHD-PAH progression remains uncertain. For example, a patient with a significant atrial septal defect may experience abnormal pulmonary flow and shear stress before meeting resting hemodynamic criteria for PAH, but current evidence does not establish whether these abnormalities predict subsequent pulmonary vascular disease. Their potential clinical value therefore requires prospective validation before shear-derived metrics can be considered for screening or treatment selection. The proposed relationships among baseline pulmonary vascular susceptibility, sustained and superimposed exposures, candidate mechanistic pathways, and clinical phenotypes are summarized conceptually in Figure 1.

## 4 Hemodynamic triggers and investigational multimodal assessment: 4D-Flow MRI and cardiopulmonary exercise testing

### 4.1 Vorticity, wall shear stress, and pulmonary flow heterogeneity

Research characterization of a hemodynamic second hit may benefit from tools capable of resolving spatial and temporal flow heterogeneity within the pulmonary vasculature, information that two-dimensional Doppler echocardiography and resting right heart catheterization do not fully capture. Relevant 4D-Flow MRI metrics for a potentially remodeling-prone pulmonary hemodynamic environment in ACHD include wall shear stress (WSS), vorticity, kinetic energy loss, and oscillatory shear patterns (46–48). In physiological conditions, pulmonary arterial flow is generally organized and energy-efficient, whereas significant shunt lesions and PAH may be associated with vortical flow, abnormal WSS distributions, and increased energy-loss indices (46, 47). Computational work suggests that such patterns may contribute to maladaptive vascular remodeling (36, 48, 49).

These abnormal flow patterns may not be confined to patients with established resting hemodynamic PAH, although the supporting evidence varies substantially across populations, as detailed below.

In adult PAH cohorts of mixed etiology, Ota et al. reviewed the application of 4D-Flow MRI in pulmonary hypertension, summarizing imaging parameters including vortical flow patterns, wall shear stress, and mean PAP estimation that provide physiological information beyond resting pressure (48). Complementary metrics including kinetic energy and energy loss have been characterized in dedicated cohorts (49, 50), with vortex duration showing a strong correlation with invasive mPAP measurements (51). These studies demonstrate physiological and measurement feasibility in pulmonary hypertension populations, but they do not establish diagnostic or prognostic utility in adults with ACHD and borderline pulmonary hemodynamics.

In a pediatric and young adult Fontan cohort, Ait Ali et al. reported associations between 4D-Flow MRI-derived kinetic energy, energy loss, flow distribution, functional status, and exercise performance (49). These findings support the physiological relevance of flow energetics in Fontan circulation but cannot be extrapolated to adults with biventricular ACHD-PAH or interpreted as validation of a screening strategy. In adults with ACHD and borderline hemodynamics, no prospective cohort has validated 4D-Flow MRI-derived WSS, vorticity, kinetic energy loss, or flow-distribution metrics as predictors of progression in patients with mildly abnormal resting hemodynamics, including mPAP 21–24 mmHg and PVR 2–3 WU. Prospective validation in this population therefore remains a research priority.

### 4.2 Investigational applications and technical limitations of 4D-Flow MRI in ACHD

Beyond flow quantification, 4D-Flow MRI has been proposed in research settings to characterize pulmonary arterial wall-related hemodynamic patterns that may be associated with pulmonary vascular dysfunction. This application has not yet been validated for clinical decision-making in ACHD. Lin et al. showed that cine MRI-derived radiomics features of the pulmonary artery correlate with hemodynamic changes in the pulmonary circulation, suggesting that structural MRI may contain hemodynamic information when analyzed with advanced post-processing (52).

These imaging signatures are relevant to the second-hit framework because they capture spatial heterogeneity in shear stress and flow energetics. Together, the mechanistic work of Shinohara et al. and the imaging observations of Lin et al. provide a rationale for testing whether advanced imaging signatures are associated with subsequent hemodynamic progression in ACHD (35, 52). Sequential 4D-Flow MRI could be evaluated as a research marker of cumulative hemodynamic exposure, but its predictive value, clinically meaningful thresholds, and ability to guide management have not been established (50, 53).

Current 4D-Flow MRI also has important technical limitations. Acquisition may require prolonged scan time and respiratory compensation, making it challenging for critically ill patients and logistically demanding in standard outpatient workflows. Temporal resolution and spatial coverage of small distal pulmonary arteries remain constrained, and vorticity quantification methods lack standardized reference values across age, sex, and CHD (53, 54). Compressed-sensing and accelerated acquisition techniques may reduce scan time, but multicenter standardization, reproducibility assessment, and ACHD-specific outcome validation are required before routine pulmonary vascular application (55).

### 4.3 Cardiopulmonary exercise testing and exercise hemodynamics

Resting hemodynamics may underestimate pulmonary vascular limitation in selected ACHD patients, particularly when pulmonary vascular reserve is impaired but resting pressures remain only mildly abnormal (12, 56–58). Some patients with normal or borderline resting pulmonary pressures may demonstrate exercise-induced pulmonary hypertension (ExPH) during provocative assessment, an entity reintroduced in the 2022 ESC/ERS guidelines and defined hemodynamically as an mPAP/cardiac output (CO) slope >3 mmHg/L/min between rest and exercise; a concurrent PAWP/CO slope >2 mmHg/L/min has been proposed to help differentiate pre- from post-capillary exercise PH (12, 58). Exercise may therefore reveal limited pulmonary vascular reserve that is not apparent under resting conditions, although this physiological observation does not establish a specific second-hit mechanism or predict progression to resting PAH (58).

Cardiopulmonary exercise testing integrated with exercise echocardiography or invasive hemodynamic measurement may provide a more comprehensive assessment of pulmonary vascular reserve (56, 57). Khattab et al. reviewed the conceptual and diagnostic validity of exercise-induced pulmonary hypertension, arguing that it represents a pathophysiologically distinct condition rather than simply an earlier stage of resting PH, while acknowledging ongoing uncertainty in threshold definitions and limitations in the available evidence (58). Combined CPET–echocardiographic assessment has identified exercise pulmonary hypertension in non-ACHD populations, including hypertrophic cardiomyopathy, but these findings provide only indirect support for application in ACHD (59). Prospective studies combining serial CPET with exercise hemodynamic assessment have not established its role in adults with ACHD and borderline resting hemodynamics (58, 60).

Among CPET-derived variables, four categories may contribute to physiological characterization in ACHD, although none is specific for early pulmonary vascular disease:

1. Peak VO_2_ is a composite measure of cardiovascular, pulmonary, and peripheral oxygen delivery. Reduced peak VO_2_ has prognostic value across ACHD populations, but it does not distinguish pulmonary vascular limitation from ventricular dysfunction, deconditioning, chronotropic impairment, or other causes of exercise intolerance (61, 62).
2. The VE/VCO_2_ slope reflects ventilatory inefficiency and carries prognostic information across several ACHD anatomies (63). An elevated value may be compatible with ventilation–perfusion mismatch or impaired pulmonary vascular efficiency, but it is not a specific diagnostic marker of ACHD-PAH.
3. End-tidal CO_2_ (PETCO_2_) provides complementary information on ventilatory and pulmonary vascular efficiency. Lower PETCO_2_ and an abnormal response during exercise have been associated with adverse outcomes in adult PAH cohorts, but PETCO_2_ remains less standardized than the VE/VCO_2_ slope and has not been validated specifically in adults with ACHD and borderline hemodynamics (64, 65).
4. Invasive cardiopulmonary exercise testing with simultaneous right heart catheterization directly characterizes exercise hemodynamics and can confirm an abnormal mPAP/CO relationship while evaluating the contribution of PAWP (12, 58). Its use is restricted largely to specialized centers. Whether iCPET improves clinical decision-making or outcomes in adults with borderline ACHD hemodynamics has not been prospectively established.

Non-invasive CPET variables may therefore identify disproportionate exercise limitation or raise suspicion of impaired pulmonary vascular reserve, whereas iCPET provides direct exercise hemodynamic measurements. No validated sequential CPET-to-iCPET pathway currently exists for screening or treatment selection in borderline ACHD-PAH.

### 4.4 Integrated CPET–4D-Flow MRI assessment: an investigational framework

The available evidence supports evaluating CPET and 4D-Flow MRI as complementary research modalities rather than as components of a validated clinical pathway. In adults with ACHD and borderline resting hemodynamics, 4D-Flow MRI could characterize spatial flow patterns and energetics, while CPET or, in selected research settings, iCPET could characterize functional and exercise hemodynamic reserve. The inclusion of these modalities within the same conceptual framework does not imply that they should be performed sequentially or that their findings should determine PAH-targeted therapy or defect closure.

**Figure 2:**
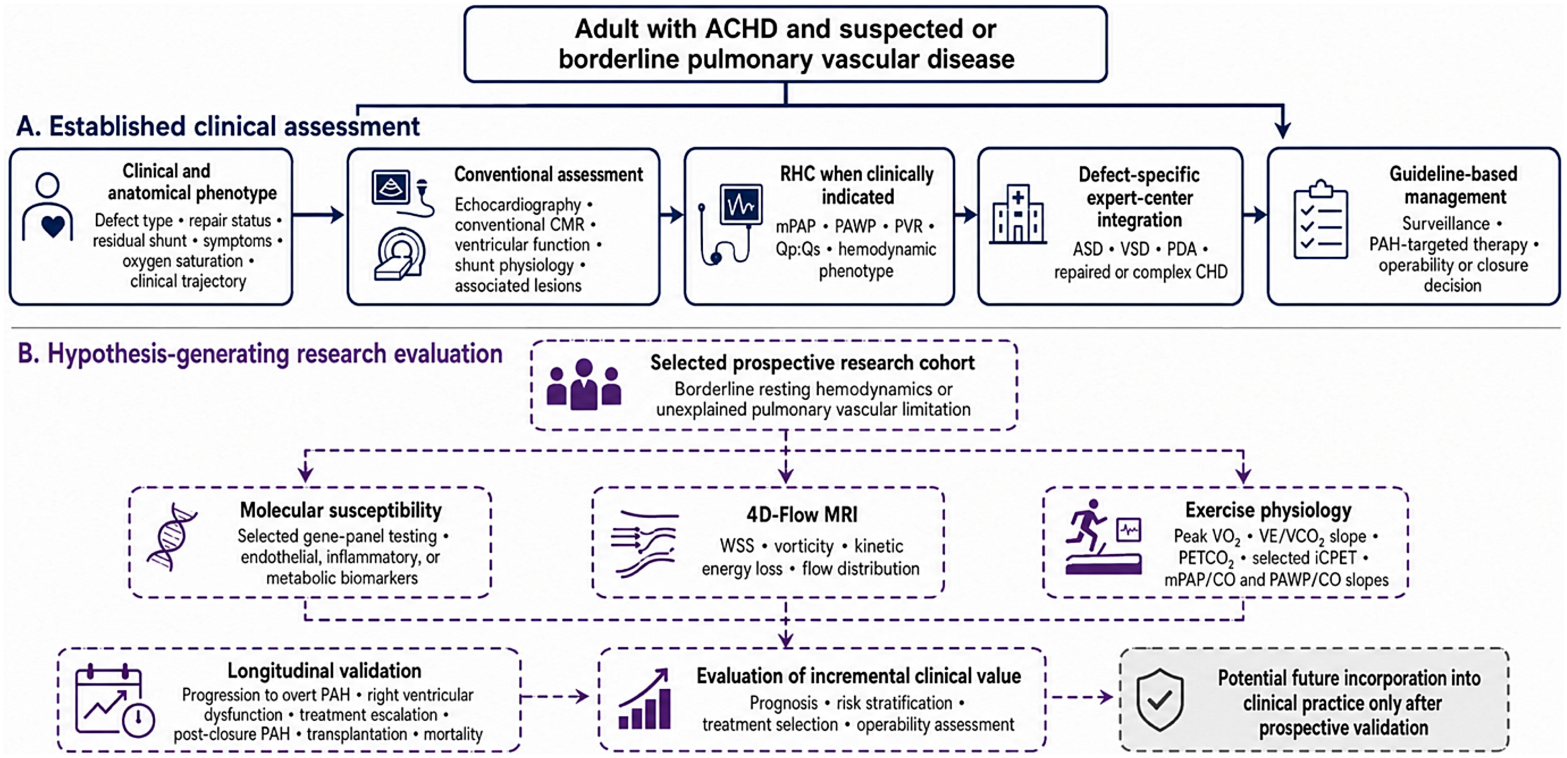
Hypothesis-generating framework distinguishing established clinical assessment from investigational evaluation in adults with ACHD and suspected or borderline pulmonary vascular disease. The upper solid-arrow pathway summarizes current expert-center assessment, including defect anatomy and repair status, conventional imaging, clinically indicated right heart catheterization, and defect-specific management. The lower dashed-border research layer depicts three parallel domains—molecular susceptibility, 4D-Flow MRI, and CPET/iCPET—that may be studied for incremental prognostic or treatment-selection value. These research domains are not intended to represent a mandatory or sequential testing pathway, and none should currently be used as a standalone criterion for PAH-targeted therapy, operability, defect closure, or treat-and-repair. Only prospective validation against clinically meaningful outcomes could support their future incorporation into clinical practice (2, 12, 48, 55–58, 66, 67). ACHD, adult congenital heart disease; ASD, atrial septal defect; CHD, congenital heart disease; CMR, cardiac magnetic resonance; CO, cardiac output; CPET, cardiopulmonary exercise testing; iCPET, invasive cardiopulmonary exercise testing; mPAP, mean pulmonary arterial pressure; PAH, pulmonary arterial hypertension; PAWP, pulmonary arterial wedge pressure; PDA, patent ductus arteriosus; PETCO_2_, end-tidal carbon dioxide; PVR, pulmonary vascular resistance; Qp:Qs, pulmonary-to-systemic blood flow ratio; RHC, right heart catheterization; VE/VCO_2_, minute ventilation to carbon dioxide production relationship; VO_2_, oxygen uptake; VSD, ventricular septal defect; WSS, wall shear stress; 4D-Flow MRI, four-dimensional flow magnetic resonance imaging.

Prospective multicenter studies are required to standardize acquisition and interpretation, establish reproducibility, and determine whether either modality provides incremental prognostic information beyond conventional anatomical assessment, echocardiography, cardiac magnetic resonance, and clinically indicated right heart catheterization. Until such evidence is available, 4D-Flow MRI and integrated CPET–4D-Flow assessment should not be used as routine screening tools or as standalone criteria for operability or treat-and-repair decisions. To clarify the distinction between established clinical assessment and investigational approaches, Table 2 summarizes their current status and evidentiary boundaries. Figure 2 presents a hypothesis-generating research framework in which molecular profiling, advanced flow imaging, and exercise physiology are evaluated as parallel research domains rather than as components of a validated sequential clinical pathway.

**Table 2:** Established clinical assessment and investigational approaches in adults with ACHD and suspected or borderline pulmonary vascular disease.

Domain	Established clinical assessment	Investigational approach or evidence gap
Clinical characterization	Longitudinal assessment at an ACHD expert center based on defect anatomy, repair status, residual lesions, symptoms, oxygen saturation, ventricular function, and clinical trajectory.	Use of genetic, endothelial, inflammatory, or metabolic profiles to predict PAH development or progression in unselected adults with ACHD and borderline hemodynamics has not been validated.
Conventional imaging	Echocardiography and conventional cardiac magnetic resonance imaging are used according to clinical indication to assess anatomy, shunt physiology, ventricular function, and associated lesions.	4D-Flow MRI-derived WSS, vorticity, kinetic energy loss, and flow-distribution metrics remain unvalidated for routine screening, prognostication, or treatment selection in ACHD-PAH.
Invasive hemodynamics	Right heart catheterization is performed when clinically indicated to confirm pulmonary hypertension, define hemodynamic phenotype, quantify PVR and shunt magnitude, and support defect-specific assessment of operability.	Exercise hemodynamics and serial iCPET have not been prospectively validated as routine tools for predicting progression or determining operability in adults with borderline ACHD hemodynamics.
Functional assessment	CPET may be used to characterize exercise capacity and provide prognostic information in ACHD, interpreted within the underlying anatomical and ventricular substrate.	Serial CPET specifically for detecting preclinical pulmonary vascular disease or guiding PAH-targeted treatment remains investigational.
Treatment and defect closure	PAH-targeted therapy, surveillance, and closure decisions are based on current guidelines, defect-specific evidence, invasive hemodynamics, and multidisciplinary expert-center assessment.	Integration of genetic testing, circulating biomarkers, 4D-Flow MRI, CPET, and iCPET as standalone or sequential criteria for PAH treatment, defect closure, or treat-and-repair candidacy has not been validated.

Investigational approaches may be appropriate within prospective research protocols or selected expert-center evaluations, but their incremental clinical value and effect on patient outcomes remain unproven. The modalities listed in the investigational column should not be interpreted as a mandatory sequential testing pathway or as standalone criteria for treatment or operability (2, 12, 48, 55–58, 66). ACHD, adult congenital heart disease; CPET, cardiopulmonary exercise testing; iCPET, invasive cardiopulmonary exercise testing; PAH, pulmonary arterial hypertension; PVR, pulmonary vascular resistance; WSS, wall shear stress; 4D-Flow MRI, four-dimensional flow magnetic resonance imaging.

## 5 Treat-and-repair: PAH-targeted therapy, operability, and defect closure

### 5.1 Eisenmenger syndrome and the limits of operability

Eisenmenger syndrome represents an advanced phenotype of shunt-associated PAH, in which progressive elevation of pulmonary vascular resistance leads to bidirectional or right-to-left shunting. Once Eisenmenger physiology is established, defect closure is generally contraindicated because eliminating the shunt may remove an important route for right-sided decompression (6, 12). Arvanitaki et al., in the 2022 JACC State-of-the-Art Review, characterized Eisenmenger syndrome as a multisystem disease extending beyond the pulmonary vasculature, with cyanosis, hematologic and coagulation abnormalities, arrhythmias, and progressive heart failure as key clinical features (6). They also emphasized that PAH-targeted therapies have improved functional status and may influence outcomes in selected patients, although advanced vascular remodeling remains largely irreversible (6, 68–73). The unifying pathophysiological substrate is advanced elevation of pulmonary vascular resistance due to pulmonary vascular remodeling, including intimal proliferation, medial hypertrophy, and plexiform lesion formation (6, 19).

Eisenmenger syndrome poses two clinical challenges. First, progression from potentially reversible pulmonary vascular disease to fixed vasculopathy may be gradual and clinically silent in its early phases. By the time resting symptoms prompt evaluation, the window for safe defect correction may have narrowed or closed. Second, current guidelines approach operability as a graded and defect-specific assessment rather than as a single universal hemodynamic cutoff (2, 12). In patients with a significant left-to-right shunt, commonly defined by Qp:Qs >1.5, closure is generally supported when PVR is <3 WU and may be considered when PVR is 3–5 WU, provided that anatomy, ventricular physiology, and the overall clinical context are favorable. When PVR exceeds 5 WU, recommendations and evidentiary support differ according to the underlying defect. In ASD, PAH-targeted therapy followed by reassessment may permit consideration of fenestrated closure if PVR decreases to <5 WU and a significant left-to-right shunt persists; complete closure is not recommended when PVR remains >5 WU. In VSD and PDA, elevated PVR requires individualized assessment in a specialized ACHD and pulmonary hypertension center, and the ASD-based treat-and-repair and fenestration experience should not be applied as an equivalent strategy (2, 12, 66). These distinctions reflect fundamental differences in pressure–flow exposure, timing of pulmonary vascular injury, and the evidence available for each anatomical substrate.

Barradas-Pires and colleagues documented that these hemodynamic thresholds originate in part from the Moller 35-year VSD cohort, which showed that a PVRi <7–8 WU·m^2^ threshold discriminated long-term survival outcomes (11). The classical INOP-study criterion, defined as PVRi <5.3 WU·m^2^ plus PVR/SVR <0.27 after inhaled nitric oxide and 100% oxygen, has been used as a historical reversibility benchmark in specialized centers. However, its supporting evidence is predominantly pediatric or mixed-age, and it should not be interpreted as a prospectively validated adult ACHD operability threshold (11).

Barradas-Pires et al. reviewed evidence that PAH-targeted therapies in Eisenmenger syndrome provide functional and hemodynamic benefit, although randomized trial data remain limited (11, 68–71, 73). Long-term outcome data from Matama et al. in a contemporary Eisenmenger cohort receiving sustained PAH-targeted therapy complement this evidence base by documenting outcomes in patients maintained on modern pharmacotherapy over extended follow-up (72). Risk stratification tools derived from the general PAH population may require recalibration for the CHD substrate. Van Dissel et al. demonstrated that a refined version of the ESC/ERS risk instrument, adjusted for CHD-specific features, improved prognostic discrimination in ACHD-PAH compared with the standard score, supporting its potential use in surveillance of patients with repaired and unrepaired shunt lesions (74).

### 5.2 PAH-targeted therapies: evidence and mechanisms in CHD

Pharmacological strategies for PAH associated with CHD broadly parallel those used for other forms of group 1 PAH and center on three major pathways: endothelin receptor antagonism, phosphodiesterase type 5 inhibition, and prostacyclin signaling (75). Mares et al. synthesized the available evidence for targeted therapies in ACHD-PAH, concluding that endothelin receptor antagonists, phosphodiesterase type 5 inhibitors, and prostacyclin analogues each have an established mechanistic rationale and accumulating evidence in this population (75). Bosentan has historically been the best-studied agent in Eisenmenger syndrome, while more recent evidence has supported sequential and, in selected patients, upfront combination strategies (68–71, 73) (Table 3). Arvanitaki and Diller reviewed the accumulated experience with PAH-targeted pharmacotherapy specifically in Eisenmenger syndrome, highlighting that functional and hemodynamic benefit extends beyond ERA monotherapy to sequential and upfront combination regimens, although randomized evidence within this population remains sparse (73).

**Table 3:** Principal PAH-targeted therapy classes relevant to ACHD-PAH and Eisenmenger syndrome.

Drug class	Representative agents	Main pathway	Clinical role in CHD-PAH	Evidence in Eisenmenger/ACHD-PAH vs. extrapolated	Key references
Endothelin receptor antagonists	Bosentan, macitentan, ambrisentan	Endothelin pathway	Bosentan is the best-studied agent in Eisenmenger syndrome; ERAs are used in symptomatic ACHD-PAH and combination strategies	Direct: BREATHE-5 (bosentan vs. placebo in Eisenmenger, RCT); MAESTRO (macitentan in Eisenmenger, RCT)	(12, 68, 70, 71, 73, 75)
Phosphodiesterase type 5 inhibitors	Sildenafil, tadalafil	Nitric oxide–cGMP pathway	Used in ACHD-PAH to improve pulmonary vascular tone, symptoms, and hemodynamics; may be part of combination therapy	Extrapolated: no Eisenmenger-specific RCT; evidence derived from idiopathic and heritable PAH trials	(12, 73, 75)
Soluble guanylate cyclase stimulators	Riociguat	Nitric oxide–sGC–cGMP pathway	Considered in selected ACHD-PAH patients with appropriate ventricular function; Eisenmenger-specific randomized data remain limited	Extrapolated: PATENT-1 enrolled group 1 PAH broadly; Eisenmenger-specific randomized data absent	(12, 75)
Prostacyclin pathway therapies	Epoprostenol, treprostinil, iloprost, selexipag	Prostacyclin pathway	Used mainly in higher-risk or refractory ACHD-PAH; ACHD-specific data are more limited than for oral therapies	Predominantly extrapolated: evidence in Eisenmenger largely observational; no Eisenmenger-specific RCT	(12, 73, 75)
Combination therapy	ERA + PDE5 inhibitor ± prostacyclin pathway therapy	Multipathway treatment	Used according to risk profile and treatment response; may support hemodynamic optimization before reassessing operability in selected patients	Extrapolated: AMBITION enrolled group 1 PAH broadly; upfront combination data in Eisenmenger remain observational	(12, 66, 73, 75)

The evidence directness column distinguishes findings derived from Eisenmenger- or ACHD-specific trials from those extrapolated from broader PAH populations. The table does not replace individualized assessment in expert centers. ACHD, adult congenital heart disease; CHD, congenital heart disease; cGMP, cyclic guanosine monophosphate; ERA, endothelin receptor antagonist; NO, nitric oxide; PAH, pulmonary arterial hypertension; ACHD-PAH, pulmonary arterial hypertension associated with congenital heart disease; PDE5, phosphodiesterase type 5; RCT, randomized controlled trial; sGC, soluble guanylate cyclase.

Direct evidence that currently available PAH-targeted therapies modify EndMT, BMPR2-related metabolism, or other remodeling pathways in ACHD-PAH has not been established. Their clinical use in this population is therefore supported primarily by functional, hemodynamic, and outcome data rather than by demonstrated reversal of the molecular mechanisms discussed above (45, 75).

Combination therapy, reflecting the multipathway nature of PAH pathogenesis, is increasingly used in expert centers according to risk profile and treatment response (12, 73, 75). The 2022 ESC/ERS guidelines recommend bosentan to improve exercise capacity in symptomatic Eisenmenger patients and supplemental iron in patients with iron deficiency (12). For patients with PAH after corrected adult CHD, initial oral combination therapy with approved PAH drugs is recommended at low and intermediate risk, while intravenous or subcutaneous prostacyclin analogues are reserved for high-risk patients (12). Sequential combination therapy is recommended in CHD-PAH patients, including those with Eisenmenger syndrome, who do not meet treatment goals. Pregnancy is contraindicated in women with Eisenmenger syndrome (12). In carefully selected patients at expert centers, PAH-targeted therapy may also be used to reduce pulmonary vascular resistance before reassessing defect operability; this forms the clinical basis of the treat-and-repair strategy.

### 5.3 The treat-and-repair strategy: reassessing operability after PAH-targeted therapy

The treat-and-repair strategy represents an approach applicable in carefully selected patients evaluated at expert centers; the available evidence does not support its generalization beyond this setting (67, 76, 77). The strategy is based on the recognition that some patients occupy an intermediate operability zone, in which PVR is elevated but may remain at least partially reversible. In these patients, resting hemodynamics may suggest high operative risk, while vasoreactivity testing, defect anatomy, clinical trajectory, and hemodynamic response to PAH-targeted therapy may still support reconsideration of closure (66). A reduction in PVR after therapy may permit reassessment of operability, but a favorable hemodynamic response does not demonstrate histological reversal of pulmonary vascular disease, establish long-term reversibility, or guarantee a favorable post-closure course (66, 67). Treat-and-repair should therefore be understood as a strategy for reassessing selected patients after medical optimization, not as a standardized pathway or proof that previously established pulmonary vasculopathy has resolved.

ASD-associated PAH. The most developed adult observational evidence for treat-and-repair concerns patients with ASD-associated PAH. Chen et al. reported an association between defect closure and favorable long-term survival in carefully selected patients, although the observational design does not establish that closure itself was responsible for the outcome (4). Li et al. studied 139 adults with ASD-PAH and reported that the TAPSE/PASP ratio and pulmonary valve peak velocity discriminated patients classified as having a potentially correctable shunt, with an internally validated area under the receiver-operating characteristic curve of 0.86 (78). This model addressed hemodynamic correctability rather than sustained response to treatment or long-term post-closure outcomes and requires external prospective validation. In selected ASD patients whose PVR decreases to <5 WU after PAH-targeted therapy and who retain a significant left-to-right shunt, current guidelines allow consideration of fenestrated closure (2, 12). Fenestration may preserve a route for right-sided decompression if pulmonary vascular load rises, but it should not be interpreted as evidence that the underlying pulmonary vasculopathy has resolved.

VSD-associated PAH. Evidence supporting treat-and-repair in VSD is substantially more limited than that available for ASD. Because VSD is a post-tricuspid shunt that exposes the pulmonary circulation to both pressure and flow loading, ASD-derived treatment-response criteria and fenestrated closure strategies cannot be assumed to apply. The available mixed-defect experience includes only small numbers of patients with VSD and does not establish a reproducible PVR threshold, treatment duration, vasoreactivity criterion, or closure technique for this population (66, 67). Decisions should therefore integrate defect size and location, shunt direction, systemic oxygen saturation, invasive hemodynamics, response to pulmonary vasodilator testing or temporary loading manipulation when appropriate, and the complete clinical trajectory within a specialized center.

PDA-associated PAH. Evidence for treat-and-repair in PDA is derived largely from small series and selected reports. Xu et al. described transcatheter PDA closure after PAH-targeted therapy in selected patients with Eisenmenger physiology (79). Such experience demonstrates that closure has been attempted in highly selected cases but does not establish general operability criteria or support routine closure in Eisenmenger syndrome. PDA produces continuous systolic and diastolic pressure–flow exposure, and its hemodynamic substrate should not be considered interchangeable with ASD or VSD. Validated criteria for partial or fenestrated PDA closure in adult PAH are lacking, and decisions require individualized invasive assessment at an expert center (12, 66, 79).

Complex CHD and repaired lesions. Evidence for treat-and-repair in complex congenital anatomy is sparse and heterogeneous. Operability cannot be inferred from thresholds or outcomes reported for isolated ASD, VSD, or PDA because previous interventions, residual lesions, ventricular physiology, collateral vessels, pulmonary artery anatomy, and unequal pulmonary blood flow may substantially alter the hemodynamic substrate (2, 12, 66, 67). Patients with PAH after previous defect correction constitute a distinct clinical group in whom anatomical repair has not prevented persistent, recurrent, or progressive pulmonary vascular disease. The term treat-and-repair should not be extended to these patients unless a clinically meaningful residual lesion remains potentially amenable to intervention.

Closure strategy and post-closure PAH. Fenestrated or partial closure has been used principally in selected patients with ASD as a means of reducing shunt burden while preserving a decompression pathway (2, 12, 66, 67, 76, 77). This strategy differs physiologically from complete closure, and standardized selection criteria and comparable long-term evidence are lacking across defect types. Successful anatomical closure does not ensure normalization of pulmonary vascular physiology. PAH may persist, recur, or progress after closure, particularly when pulmonary vascular disease was established before repair (1, 5, 12, 66, 67). Post-closure assessment should therefore include continued follow-up in an ACHD and pulmonary hypertension program, with reassessment of symptoms, oxygen saturation, exercise capacity, right ventricular function, and hemodynamics when clinically indicated. Treat-and-repair should be interpreted as reassessment of operability after therapy, not as proof of vascular cure.

No validated model currently predicts which patients will achieve a sustained hemodynamic response or a favorable post-closure outcome. Patient selection remains based on multidisciplinary expert-center assessment of defect anatomy, invasive hemodynamics, shunt magnitude and direction, systemic oxygen saturation, clinical trajectory, and response to therapy (66, 67, 76, 77). Future prospective studies should report results separately for ASD, VSD, PDA, and complex CHD rather than combining anatomically and physiologically distinct lesions into a single treat-and-repair population.

## 6 Extended application of the framework: Fontan as a low-flow, low-reserve pulmonary vascular state

### 6.1 Hemodynamic basis of low-flow, low-reserve vulnerability

Within the second-hit framework, Fontan physiology occupies a distinct position from classic shunt-associated PAH. Fontan circulation should not be classified as a classical high-pressure ACHD-PAH phenotype because pulmonary blood flow occurs without a subpulmonary ventricular pump and depends on passive venous-to-atrial pressure gradients (80, 81). Even modest increases in PVR or losses of pulmonary vascular compliance may impair transpulmonary flow, reduce ventricular preload, and increase systemic venous pressure (80–82). Fontan physiology is therefore considered here as a model of low-flow, low-reserve pulmonary vascular vulnerability rather than as an extension of Eisenmenger pathophysiology.

The consequences of this low-reserve state extend beyond the pulmonary circulation. Chronic systemic and hepatic venous congestion contributes to Fontan-associated liver disease, which may progress from sinusoidal congestion and cellular stress to fibrosis and cirrhosis (83–85). These hepatic complications reflect the sustained Fontan hemodynamic substrate and should not be interpreted as downstream evidence of the same pulmonary vascular remodeling sequence proposed for shunt-associated PAH.

Elevated venous and lymphatic pressures may also contribute to protein-losing enteropathy and plastic bronchitis (86–88). These complications illustrate how a limited pulmonary vascular reserve can interact with systemic venous and lymphatic vulnerability, but their pathogenesis is multifactorial and cannot be attributed solely to increased PVR. The Fontan circulation therefore broadens the conceptual relevance of cumulative hemodynamic stress while remaining pathophysiologically distinct from classical ACHD-PAH.

### 6.2 Clinical implications and limits of extrapolation

Pulmonary vascular load and systemic venous congestion are closely linked in Fontan circulation. An increase in pulmonary vascular impedance may reduce pulmonary blood flow and ventricular preload while increasing upstream venous and lymphatic pressure; conversely, ventricular dysfunction, atrioventricular valve disease, arrhythmia, or advanced end-organ dysfunction may further destabilize the circulation (80–82, 89). The resulting phenotype is therefore a circulatory and multiorgan syndrome rather than an isolated pulmonary arterial disease.

Pulmonary vasodilator therapies, including phosphodiesterase type 5 inhibitors and endothelin receptor antagonists, have been used in selected patients with Fontan circulation, particularly when impaired pulmonary vascular reserve is suspected (82, 89). However, the evidence is heterogeneous, and these therapies should not be presented as routine treatment for all Fontan patients or as evidence that Fontan failure represents classical PAH. Direct evidence that lowering pulmonary vascular load prevents progression of Fontan-associated liver disease, protein-losing enteropathy, or lymphatic complications remains limited (82, 86–89).

Fontan failure may follow different trajectories involving impaired ventricular function, elevated venous pressure, reduced cardiac output, arrhythmia, lymphatic failure, or end-organ disease (80–82, 89, 90). Within this review, Fontan circulation serves only as an extended example of how limited pulmonary vascular reserve can interact with cumulative hemodynamic and organ-specific vulnerability. It is not included as an anatomical subtype of treat-and-repair, a classical ACHD-PAH phenotype, or evidence of the same causal sequence proposed for Eisenmenger syndrome.

## 7 Discussion

### 7.1 The second hit as an integrative framework

Genetic susceptibility, defect-specific hemodynamic exposure, and treat-and-repair evidence converge on a common interpretation: PAH in ACHD is not determined by anatomy alone but by interactions among congenital substrate, pulmonary vascular susceptibility, and superimposed stressors over time. This framework may help explain the divergent pulmonary vascular trajectories observed among patients with anatomically similar defects. Fontan physiology is considered separately as a low-flow, low-reserve state rather than as a classical ACHD-PAH phenotype (Section 6).

Converging data from genetic, mechanobiological, and computational studies support the view that PAH susceptibility reflects a systems-level vulnerability involving vascular tone, cellular metabolism, endothelial identity, and remodeling capacity (17, 19, 22, 29). Abnormal shear stress in post-tricuspid defects may contribute to these processes, but the supporting evidence is largely experimental or computational (34, 35). These data do not establish a causal sequence in logitudinal ACHD cohorts.

The principal translational gap is the absence of prospective studies connecting this mechanistic framework to clinical detection. Serial 4D-Flow MRI and CPET-based exercise hemodynamics are positioned to test whether imaging- or exercise-detected abnormalities precede hemodynamic progression in adults with borderline ACHD-associated pulmonary vascular disease, but neither has been prospectively validated in this population (12, 46–48, 56–60). Across the four domains reviewed, the evidence is not uniform in its clinical maturity: genetic susceptibility and PAH-targeted therapy before defect reassessment are supported by more clinically developed evidence, with established guidelines and accumulating observational data, whereas hemodynamic imaging with 4D-Flow MRI, exercise-based assessment with CPET, and investigational biomarkers remain hypothesis-generating and require prospective validation in ACHD-specific cohorts before their role in clinical practice can be defined.

### 7.2 Graded confidence across domains

The asymmetry in evidence maturity identified across the four domains should guide clinical interpretation and research priorities, with genetic susceptibility and PAH-targeted therapy representing the most clinically developed areas and hemodynamic imaging and exercise testing remaining investigational.

Evidence for genetic susceptibility is gene-specific. BMPR2 haploinsufficiency is experimentally supported as a determinant of vulnerability to hemodynamic stress (19, 21, 22), and SOX17 carries strong gene-disease validity as a PAH susceptibility gene, with SOX17-associated PAH occurring frequently in the setting of congenital cardiac anomalies (14, 15, 23–25). The interaction of these genetic factors with sex-related and metabolic pathways may further modify disease expression (14, 25). Genetic counseling for relatives of patients with pathogenic PAH variants is recommended by current guidelines, though whether similar approaches should extend to asymptomatic ACHD patients with borderline hemodynamics remains unresolved (12).

Eisenmenger management and treat-and-repair are supported by accumulating but largely observational evidence. PAH-targeted therapies have improved functional status in Eisenmenger syndrome, though whether they modify long-term vascular disease progression remains uncertain (6, 68–73). Operability is best approached as a graded, defect-specific assessment in which PVR reversibility, defect anatomy, and clinical trajectory inform closure decisions more reliably than resting hemodynamics alone (11, 66); the available evidence does not support uniform application of closure criteria across ASD, VSD, and PDA. Preliminary echocardiographic models incorporating TAPSE/PASP ratio and pulmonary valve peak velocity show discriminatory value for correctable shunts in selected ASD-PAH cohorts, though prospective validation remains lacking (78). These data are directionally supportive but derive predominantly from observational series and single-center cohorts, and randomized evidence evaluating treat-and-repair in ACHD-PAH is absent (67, 76–78).

Hemodynamic imaging and exercise testing represent the domain with the greatest distance between mechanistic rationale and clinical validation. The rationale for 4D-Flow MRI surveillance is coherent, as pathological shear may promote EndMT, computational models capture maladaptive remodeling transitions, and flow parameters are quantifiable non-invasively (35, 36, 48, 55), but the critical prospective study linking imaging-detected abnormalities to hemodynamic progression in borderline ACHD has not been performed. CPET occupies a parallel position: the physiological rationale for exercise-based unmasking of limited pulmonary vascular reserve is supported by current guidelines (12, 58), but ACHD-specific prospective data remain insufficient to define its role in early PAH risk stratification (56, 57, 60). Investigational biomarkers relevant to endothelial dysfunction, vascular remodeling, and metabolic reprogramming have been proposed as potential tools for earlier disease detection in PAH, but their validation in ACHD-specific cohorts remains limited and their clinical role undefined (17, 19, 44). Accordingly, these approaches remain investigational rather than practice-defining.

### 7.3 Sex as a modifying variable

Sex may modify several components of the second-hit framework, though this dimension of ACHD-PAH susceptibility has received limited prospective evaluation. Experimental and clinical data indicate that SOX17 expression is subject to sex-related regulation, with estrogen metabolites implicated in its transcriptional repression and differential expression reported across sexes in PAH (14, 25). However, the relevance of these observations to sex-specific susceptibility in ACHD-PAH remains uncertain. Whether sex should inform sex-stratified screening, surveillance, or therapeutic strategies in ACHD-PAH remains uncertain and requires prospective evaluation in ACHD-specific cohorts.

### 7.4 Pediatric CHD-PAH and the limits of translation to adult disease

Pediatric CHD-associated pulmonary hypertension differs from ACHD-PAH in both biological context and clinical trajectory. In infants and children, pulmonary vascular disease may develop during ongoing lung and vascular maturation and may be influenced by prenatal factors, prematurity, developmental lung disease, genetic syndromes, and exposure to abnormal pulmonary flow or pressure from early life (91, 92). The interval between hemodynamic exposure and clinically important vascular disease may therefore be shorter than in adults, and decisions regarding the timing of repair are closely linked to pulmonary vascular development rather than solely to adult hemodynamic thresholds.

Pediatric cohorts also include a higher relative contribution of developmental and syndromic conditions. T-box-related variants, trisomy 21, abnormal lung development, and complex congenital anatomy may modify pulmonary vascular susceptibility and operability (27, 91–94). These observations support the biological plausibility of early susceptibility but should not be translated directly into assumptions about genetic risk, reversibility, or treatment response in adults who have survived for decades with repaired or unrepaired lesions. Conversely, adult ACHD-PAH reflects cumulative exposure, previous interventions, residual lesions, ventricular adaptation, acquired comorbidities, and survivor selection, factors that are not fully represented in pediatric studies.

Evidence for PAH-targeted therapy in children is also less extensive and frequently relies on pediatric observational cohorts, pharmacokinetic experience, or extrapolation from adult PAH trials (91, 92, 95, 96). Pediatric evidence may inform developmental mechanisms, high-risk phenotypes, and the importance of early assessment, but it should be classified as indirect when supporting adult ACHD-PAH treatment, operability thresholds, or treat-and-repair decisions. Future longitudinal studies linking pediatric exposure and treatment history with adult pulmonary vascular outcomes may help determine which early-life factors influence later ACHD-PAH susceptibility.

### 7.5 Clinical relevance and research priorities

The second-hit framework highlights three potential clinical areas: genetic risk identification before overt hemodynamic compromise, detection of abnormal hemodynamic stress before advanced structural remodeling, and reduction of PVR before a defect can no longer be safely closed. The first and third checkpoints are partially supported by current evidence and guidelines. Gene-panel diagnostics can identify at-risk individuals in selected PAH contexts (15, 20, 24), and treat-and-repair strategies attempt to use the PVR-reversibility window before reassessing defect closure (66, 67, 76, 77), though both applications require careful interpretation given the incomplete evidence base and phenotype-dependent patient selection in ACHD.

The second area, identifying patients in whom hemodynamic stress may be contributing to early pulmonary vascular remodeling, is the least clinically validated. 4D-Flow MRI and exercise hemodynamics have been proposed as research tools for examining this concept, but prospective ACHD-specific data are not yet sufficient to define their role as screening or treatment-selection tools (12, 48, 56–58). At present, their role is best viewed as investigational rather than practice-defining.

Several components of the evidence reviewed are derived partly from pediatric or mixed-age populations, including operability criteria (11), T-box-related susceptibility (27, 28), and pulmonary vascular vulnerability associated with trisomy 21 (93). These findings are relevant to the broader congenital heart disease context but remain indirect when applied to adults with ACHD-PAH.

The principal research priorities follow directly from these evidence gaps. Prospective studies should determine whether genetic susceptibility, advanced flow imaging, and exercise physiology provide incremental prognostic information beyond established clinical assessment in adults with borderline ACHD hemodynamics. Their inclusion within the same conceptual framework should not be interpreted as an established sequential testing pathway. Treat-and-repair requires multicenter prospective studies with defect-specific enrollment, prespecified hemodynamic response criteria, standardized closure strategies, and long-term assessment of persistent or recurrent PAH after closure. Pediatric-to-adult longitudinal cohorts are also needed to determine how early-life hemodynamic exposure, genetic syndromes, timing of repair, and pulmonary vascular development influence later ACHD-PAH susceptibility (27, 28, 48, 56–58, 66, 67, 78, 91, 92, 94).

### 7.6 Limitations

Several methodological limitations of this review should be acknowledged. The literature search was restricted to PubMed and prioritized publications from 2019 to 2026, with selected earlier publications retained when considered foundational for molecular, genetic, mechanistic, or clinical context. This approach may have underrepresented conference-reported data, non-English publications, and studies indexed outside MEDLINE. As a narrative review, no formal risk-of-bias assessment, certainty grading, meta-analytic pooling of effect estimates, or fully reproducible search strategy was performed; the selection and synthesis of evidence reflects editorial judgment rather than a prespecified systematic protocol. The narrative design also introduces the possibility of selective inclusion of studies that support the second-hit framework, which may overrepresent mechanistically coherent evidence relative to null or contradictory findings.

The included literature spans pre-tricuspid shunts, post-tricuspid defects, repaired lesions, Eisenmenger physiology, and single-ventricle Fontan circulation. Many studies also include pediatric populations, mixed congenital heart disease cohorts, non-ACHD PAH populations, Fontan physiology, or experimental and computational models, each of which differs from the adult ACHD-PAH population in hemodynamic substrate, vascular biology, or clinical context, limiting the directness of inference to adults with ACHD-PAH. The inclusion of Fontan data further increases this heterogeneity because Fontan physiology is considered here as a distinct low-flow, low-reserve state rather than a classical ACHD-PAH phenotype. The evidence base for treat-and-repair, 4D-Flow MRI assessment, and serial CPET in adults with borderline hemodynamic ACHD remains predominantly observational, and prospective validation for prediction, patient selection, and clinical decision making is lacking. Finally, the second-hit framework synthesized here is compatible with the reviewed evidence but has not been prospectively validated as a causal sequence in longitudinal ACHD cohorts. Its value therefore lies in organizing heterogeneous evidence and clarifying areas of uncertainty, rather than in providing an established clinical algorithm.

## 8 Conclusions

The evidence reviewed is compatible with, but does not establish, a second-hit model in which baseline pulmonary vascular susceptibility interacts with sustained or superimposed hemodynamic, hypoxemic, inflammatory, metabolic, or clinical stressors in ACHD. The directness of this evidence varies substantially. SOX17 has a direct association with ACHD-PAH, whereas much of the evidence involving BMPR2-related signaling, endothelial-to-mesenchymal transition, metabolic reprogramming, and mechanotransduction is derived from heritable PAH, broader PAH populations, or experimental and computational models. PAH-targeted therapy and guideline-based, defect-specific assessment of operability represent established clinical domains, but treat-and-repair remains supported predominantly by observational evidence from selected expert-center populations. Four-dimensional flow MRI, exercise hemodynamics, and their integration with genetic or biomarker data remain investigational and have not been validated for screening or treatment selection in adults with borderline ACHD hemodynamics.

The principal contribution of this narrative review is to organize heterogeneous evidence according to its clinical and biological directness, clarify the distinction between established management and hypothesis-generating approaches, and identify priorities for prospective research. These priorities include longitudinal evaluation of pulmonary vascular susceptibility in ACHD, validation of imaging and exercise-based markers of progression, and multicenter assessment of defect-specific treat-and-repair strategies and post-closure outcomes. Fontan circulation should be interpreted separately as a low-flow, low-reserve state rather than as classical high-pressure ACHD-PAH. Accordingly, the second-hit framework represents a conceptual synthesis and research agenda, not a prospectively validated causal sequence or clinical algorithm.
