# Supplementary material for: A second-hit conceptual framework for pulmonary arterial hypertension in adult congenital heart disease: genetics, hemodynamics, and treat-and-repair
Source: Front Cardiovasc Med. 2026 Aug 4;13:1907009. doi: 10.3389/fcvm.2026.1907009 (PMC13482457; doi:10.3389/fcvm.2026.1907009)
Supplement: Supplementary file 1 [file Supplementaryfile1.docx]

**Supplementary Material**

**A Second-Hit Framework for Pulmonary Arterial Hypertension in Adult Congenital Heart Disease: Genetics, Hemodynamics, and Treat-and-Repair**

**Supplementary Methods**

This supplementary methods appendix describes the structured, purposive literature-search framework used to support the narrative review. The review was designed as an evidence-informed narrative synthesis rather than a systematic review. Its purpose was to integrate genetic, endothelial, hemodynamic, imaging-based, therapeutic, and multiorgan evidence into a conceptual second-hit framework for adult congenital heart disease-associated pulmonary arterial hypertension (ACHD-PAH), rather than to estimate pooled treatment effects, perform diagnostic test accuracy synthesis, or produce certainty ratings.

A systematic-review protocol was not registered, a PRISMA flow diagram was not constructed, and duplicate independent screening, exhaustive database coverage, formal risk-of-bias assessment, certainty grading, and quantitative meta-analysis were not performed. These procedures were not undertaken because the review question was deliberately broad, mechanistic, and integrative, spanning clinical guidelines, observational ACHD cohorts, molecular and experimental studies, imaging studies, pharmacological evidence, Fontan physiology, and expert synthesis. The search strategy was therefore used to identify high-relevance peer-reviewed sources for narrative integration, not to enumerate all eligible records or generate reproducible study counts.

The manuscript was prepared with reference to reporting principles reflected in the Scale for the Assessment of Narrative Review Articles (SANRA), particularly justification of the review’s importance, clear statement of aims, transparent description of the literature-search approach, appropriate referencing, and scientific reasoning. However, no formal item-level SANRA scoring was performed, and the review should not be represented as a completed SANRA-scored assessment.

A structured PubMed search was conducted in April 2026. PubMed was selected as the primary database because it provides broad biomedical indexing of pulmonary vascular disease, congenital heart disease, cardiovascular imaging, exercise physiology, and clinical therapeutics. The search combined Medical Subject Headings (MeSH) and free-text terms across four prespecified thematic domains: genetic susceptibility and endothelial biology; multimodal hemodynamic and exercise-based assessment; PAH-targeted therapy and defect operability; and Fontan physiology with systemic complications. Terms within each domain were combined using OR, and domains were combined using AND when cross-domain specificity was required. Search strings were refined iteratively to improve thematic relevance and identify clinically and mechanistically informative studies.

English-language peer-reviewed publications from 2019 to 2026 were prioritized. Select foundational references from 2018-2020 and earlier seminal guideline or mechanistic sources were retained when required for clinical definitions, operability thresholds, PAH genetics, congenital heart disease context, or mechanistic interpretation. Preprints and non-peer-reviewed sources were excluded. Eligible source types included clinical guidelines, consensus statements, randomized trials when available, prospective and retrospective observational studies, registry analyses, systematic reviews, authoritative narrative reviews, and mechanistic, experimental, or computational studies relevant to the second-hit framework.

Articles were selected purposively according to thematic relevance, methodological credibility, clinical applicability, and contribution to the conceptual framework. Evidence was interpreted according to directness to ACHD-PAH. Direct evidence referred to adult congenital heart disease populations with PAH, Eisenmenger syndrome, shunt-associated PAH, repaired congenital lesions, or Fontan physiology. Partially direct evidence included mixed-age or mixed-congenital-heart-disease cohorts where ACHD-PAH-relevant findings could be interpreted but were not isolated. Indirect evidence included broader PAH cohorts, pediatric CHD/PAH cohorts, experimental or computational studies, and non-ACHD populations used to support biological plausibility. Guidelines, adult ACHD cohorts, prospective studies, large observational series, and systematic reviews were given greater interpretive weight when available. Mechanistic, pediatric, computational, and imaging-based evidence was used primarily to support plausibility, identify knowledge gaps, and generate hypotheses rather than to establish clinical recommendations.

Because record counts were not prospectively logged, the authors do not report numbers of retrieved, screened, excluded, or included records. The absence of record enumeration is consistent with the narrative design but should be acknowledged as a limitation. To improve transparency, the search domains, eligibility principles, evidence-directness categories, and representative PubMed query structures are provided below.

**Supplementary Table S1. Thematic search domains, MeSH terms, and free-text terms used to support the narrative review.**

| **Thematic domain** | **MeSH terms** | **Free-text terms** |
| --- | --- | --- |
| 1. Genetic susceptibility and endothelial biology | "Hypertension, Pulmonary"[MeSH]; "Heart Defects, Congenital"[MeSH]; "Endothelial-Mesenchymal Transition"[MeSH]; "Endothelium, Vascular"[MeSH]; "Genetic Predisposition to Disease"[MeSH] | "SOX17"; "BMPR2"; "TBX4"; "FOXF1"; "KCNK3"; "endothelial-to-mesenchymal transition"; "EndMT"; "pulmonary arterial hypertension"; "congenital heart disease"; "adult congenital heart disease"; "PAH-CHD"; "ACHD-PAH"; "second hit"; "vascular remodeling"; "shear stress" |
| 2. Multimodal hemodynamic and exercise-based assessment | "Magnetic Resonance Imaging"[MeSH]; "Exercise Test"[MeSH]; "Hemodynamics"[MeSH]; "Cardiac Catheterization"[MeSH]; "Vascular Resistance"[MeSH] | "4D-Flow cardiac magnetic resonance"; "four-dimensional flow magnetic resonance imaging"; "4D-flow MRI"; "wall shear stress"; "vorticity"; "oscillatory shear"; "kinetic energy loss"; "cardiopulmonary exercise testing"; "CPET"; "invasive CPET"; "exercise hemodynamics"; "exercise-induced pulmonary hypertension"; "pulmonary hypertension screening"; "right heart catheterization" |
| 3. PAH-targeted therapy and defect operability | "Eisenmenger Complex"[MeSH]; "Antihypertensive Agents"[MeSH]; "Cardiac Surgical Procedures"[MeSH]; "Heart Septal Defects"[MeSH]; "Vasodilator Agents"[MeSH] | "Eisenmenger syndrome"; "pulmonary arterial hypertension-targeted therapy"; "PAH-targeted therapy"; "treat-and-repair"; "treat and repair"; "defect closure"; "shunt closure"; "operability"; "atrial septal defect"; "ventricular septal defect"; "patent ductus arteriosus"; "pulmonary vascular resistance"; "PVR"; "endothelin receptor antagonist"; "phosphodiesterase type 5 inhibitor"; "prostacyclin" |
| 4. Fontan physiology and systemic complications | "Fontan Procedure"[MeSH]; "Protein-Losing Enteropathies"[MeSH]; "Vascular Resistance"[MeSH]; "Liver Cirrhosis"[MeSH]; "Lymphatic Diseases"[MeSH] | "Fontan circulation"; "failing Fontan"; "Fontan-associated liver disease"; "FALD"; "protein-losing enteropathy"; "plastic bronchitis"; "lymphatic dysfunction"; "pulmonary vascular resistance"; "central venous pressure"; "hepatic congestion"; "heart-liver transplantation" |

Note: Terms within domains were combined with OR. Domains were combined with AND when the search required cross-domain specificity. The list reflects the structured search framework used to identify relevant literature for narrative synthesis rather than a locked systematic-review strategy.

**Supplementary Table S2. Eligibility and relevance criteria used for purposive source selection.**

| **Criterion** | **Included / prioritized** | **Excluded / deprioritized** |
| --- | --- | --- |
| Population or model | Adult congenital heart disease populations with PAH, Eisenmenger syndrome, shunt-associated PAH, repaired congenital lesions, or Fontan physiology. Broader PAH cohorts, pediatric CHD/PAH cohorts, experimental models, and computational models were eligible when they supported biological plausibility or helped define research gaps. | Studies unrelated to pulmonary vascular disease, congenital heart disease, Fontan physiology, PAH genetics, hemodynamic imaging, exercise hemodynamics, or PAH-targeted therapy. |
| Conceptual relevance | Sources addressing genetic susceptibility, endothelial dysfunction, EndMT, shear stress, 4D-flow MRI, CPET, exercise hemodynamics, PAH therapy, operability, treat-and-repair, Fontan failure, FALD, PLE, or lymphatic complications. | Sources with no clear contribution to the second-hit framework or to clinical interpretation of ACHD-PAH. |
| Publication type | Clinical guidelines, consensus statements, randomized trials when available, prospective and retrospective observational studies, registry analyses, systematic reviews, authoritative narrative reviews, mechanistic studies, experimental studies, and computational hemodynamic studies. | Preprints, non-peer-reviewed materials, conference abstracts without full peer-reviewed publication, letters or editorials without substantial evidentiary or conceptual contribution. |
| Date range | English-language peer-reviewed publications from 2019-2026 were prioritized. Foundational references from 2018-2020 and selected earlier guideline or seminal mechanistic sources were retained when essential for context. | Older sources were generally not prioritized unless they were foundational or guideline-defining. |
| Language | English-language publications. | Non-English publications were not included. |
| Selection logic | Purposive selection based on thematic relevance, methodological credibility, clinical applicability, and contribution to the conceptual synthesis. | No claim was made that all eligible publications were captured or that selection was exhaustive. |

Note: These criteria were applied to support a transparent narrative review. They were not used as a duplicate-screened systematic-review eligibility framework.

**Supplementary Table S3. Evidence-directness categories and interpretive weighting.**

| **Evidence category** | **Typical source type** | **Interpretive use** | **Examples of manuscript use** |
| --- | --- | --- | --- |
| Direct ACHD-PAH evidence | Adult CHD patients with PAH; Eisenmenger syndrome; shunt-associated PAH; repaired congenital lesions with PAH; adult Fontan cohorts when pulmonary vascular load or Fontan failure was directly relevant. | Highest interpretive weight for ACHD-PAH-specific clinical claims. Used to support clinically grounded statements when available. | Operability, PAH-CHD risk stratification, Eisenmenger therapy, adult ACHD outcomes. |
| Partially direct CHD/PAH evidence | Mixed adult/pediatric CHD-PAH cohorts; mixed CHD populations where ACHD-specific data were not fully isolated; Fontan cohorts including adolescents/young adults. | Moderate interpretive weight. Used cautiously, with explicit acknowledgement of limited adult ACHD specificity. | Fontan flow energetics, pediatric-to-adult extrapolation, mixed CHD pharmacotherapy evidence. |
| Indirect PAH evidence | Idiopathic PAH, heritable PAH, broader group 1 PAH cohorts, non-CHD PAH cohorts, or PAH genetics studies not restricted to ACHD. | Used to support pathobiology, pharmacological rationale, genetics, or external validity; not used alone to make ACHD-specific clinical recommendations. | BMPR2 pathways, broader PAH risk tools, CPET/ExPH evidence outside ACHD. |
| Mechanistic / experimental / computational evidence | Cellular, animal, molecular, endothelial, EndMT, shear-stress, and computational hemodynamic studies. | Used primarily for biological plausibility and hypothesis generation. Clinical claims were framed as unvalidated unless supported by human ACHD data. | Second-hit biology, mechanotransduction, EndMT, shear-stress modeling. |
| Contextual expert synthesis | Authoritative reviews, state-of-the-art reviews, clinical commentaries, and expert consensus documents. | Used to frame knowledge gaps, clinical reasoning, and terminology. Not treated as primary causal evidence. | Narrative framing of Eisenmenger syndrome, failing Fontan, treat-and-repair rationale. |

Note: Evidence directness was considered when wording conclusions. Mechanistic and extrapolated evidence was described as biologically plausible or hypothesis-generating unless supported by direct ACHD-PAH data.

**Supplementary Table S4. SANRA-informed reporting transparency statement.**

| **SANRA-related reporting domain** | **How this review addressed the domain** | **Important caveat** |
| --- | --- | --- |
| Importance for readership | The review addresses the clinical heterogeneity of ACHD-PAH and proposes an integrative second-hit framework linking susceptibility, hemodynamic stress, therapy, and multiorgan consequences. | This is a narrative rationale, not a systematic-review question framed by PICO. |
| Statement of aims | The manuscript states that it aims to provide an integrated narrative review of ACHD-PAH and identify clinical checkpoints and research priorities. | Aims are conceptual and translational rather than designed to estimate effect sizes. |
| Description of literature search | The search database, date, thematic domains, terms, language restriction, date range, excluded preprints, and iterative Boolean approach are described in the manuscript and this supplement. | Record counts were not prospectively logged; no PRISMA flow diagram is provided. |
| Referencing | Clinical guidelines, ACHD cohorts, PAH genetics, mechanistic studies, imaging studies, therapy literature, and Fontan literature are cited throughout the manuscript. | Narrative selection can still introduce selection bias, which is acknowledged as a limitation. |
| Scientific reasoning | Evidence is interpreted according to directness to ACHD-PAH, and extrapolated/mechanistic evidence is framed as hypothesis-generating. | No formal certainty grading or risk-of-bias tool was applied. |
| Appropriate presentation of data | Tables and sections distinguish direct, indirect, and extrapolated evidence; 4D-flow MRI and CPET are presented as research priorities rather than established screening tools. | The review should not be presented as a SANRA-scored review, PRISMA review, or systematic review. |

Note: SANRA was used as a reporting reference for narrative-review transparency. The authors did not perform formal item-level SANRA scoring.

**Supplementary Table S5. Representative PubMed query structures used iteratively during the search.**

| **Search focus** | **Representative PubMed query structure** |
| --- | --- |
| Genetics / endothelial biology | (("Hypertension, Pulmonary"[MeSH] OR "pulmonary arterial hypertension" OR PAH) AND ("Heart Defects, Congenital"[MeSH] OR "congenital heart disease" OR "adult congenital heart disease" OR ACHD OR "PAH-CHD") AND (SOX17 OR BMPR2 OR TBX4 OR FOXF1 OR KCNK3 OR "endothelial-to-mesenchymal transition" OR EndMT OR "vascular remodeling")) |
| 4D-flow MRI / hemodynamics | (("Hypertension, Pulmonary"[MeSH] OR "pulmonary hypertension" OR "pulmonary arterial hypertension") AND ("Magnetic Resonance Imaging"[MeSH] OR "4D-flow MRI" OR "four-dimensional flow magnetic resonance" OR "wall shear stress" OR vorticity OR "kinetic energy loss") AND ("congenital heart disease" OR ACHD OR Fontan OR "pulmonary vascular resistance")) |
| CPET / exercise hemodynamics | (("Exercise Test"[MeSH] OR "cardiopulmonary exercise testing" OR CPET OR "invasive CPET" OR "exercise hemodynamics") AND ("pulmonary hypertension" OR "exercise-induced pulmonary hypertension" OR "pulmonary vascular reserve") AND ("congenital heart disease" OR ACHD OR Fontan)) |
| Treat-and-repair / operability | (("Eisenmenger Complex"[MeSH] OR Eisenmenger OR "PAH-CHD" OR "shunt-associated PAH") AND ("PAH-targeted therapy" OR bosentan OR macitentan OR sildenafil OR tadalafil OR prostacyclin OR "endothelin receptor antagonist" OR "phosphodiesterase type 5 inhibitor") AND ("treat-and-repair" OR "defect closure" OR "shunt closure" OR operability OR "pulmonary vascular resistance")) |
| Fontan / systemic complications | (("Fontan Procedure"[MeSH] OR "Fontan circulation" OR "failing Fontan") AND ("pulmonary vascular resistance" OR "central venous pressure" OR "Fontan-associated liver disease" OR FALD OR "protein-losing enteropathy" OR "plastic bronchitis" OR "lymphatic dysfunction")) |

Note: Query structures are provided for transparency. Searches were refined iteratively based on yield and relevance, so these examples should be interpreted as representative structures rather than a closed systematic-review search log.

**Supplementary limitations of the search approach**

The search strategy was restricted to PubMed and English-language peer-reviewed publications. Databases such as Embase, Scopus, Web of Science, and Cochrane Library were not searched. Grey literature, conference abstracts without full manuscripts, and preprints were not included. Because retrieved and excluded records were not prospectively enumerated, the search cannot support PRISMA-style reporting, reproducible record counts, or quantitative conclusions about the total evidence base. These limitations are consistent with the narrative-review design and should be acknowledged in the manuscript.
